# Long-term efficacy and safety of certolizumab pegol in Japanese rheumatoid arthritis patients with an inadequate response to methotrexate: 52-week results from an open-label extension of the J-RAPID study

**DOI:** 10.3109/14397595.2014.881709

**Published:** 2014-03-04

**Authors:** Yoshiya Tanaka, Kazuhiko Yamamoto, Tsutomu Takeuchi, Hisashi Yamanaka, Naoki Ishiguro, Katsumi Eguchi, Akira Watanabe, Hideki Origasa, Toshiharu Shoji, Nobuyuki Miyasaka, Takao Koike

**Affiliations:** ^a^ First Department of Internal Medicine, University of Occupational and Environmental Health, Japan, Kitakyushu, Japan; ^b^ Department of Allergy and Rheumatology, Graduate School of Medicine, The University of Tokyo, Bunkyo-ku, Tokyo, Japan; ^c^ Division of Rheumatology, Department of Internal Medicine, Keio University School of Medicine, Shinjuku-ku, Tokyo, Japan; ^d^ Institute of Rheumatology, Tokyo Women's Medical University, Shinjuku-ku, Tokyo, Japan; ^e^ Department of Orthopedic Surgery, Nagoya University Graduate School and Faculty of Medicine, Nagoya, Aichi, Japan; ^f^ Sasebo City General Hospital, Sasebo, Nagasaki, Japan; ^g^ Research Division for Development of Anti-Infectious Agents, Institute of Development, Aging and Cancer, Tohoku University, Sendai, Miyagi, Japan; ^h^ Division of Biostatistics and Clinical Epidemiology, University of Toyama School of Medicine, Toyama, Toyama, Japan; ^i^ Department of Clinical Research and Development, UCB, Shinjuku-ku, Tokyo, Japan; ^j^ Department of Medicine and Rheumatology, Graduate School of Medicine and Dentistry, Tokyo Medical and Dental University, Bunkyo-ku, Tokyo, Japan; ^k^ NTT Sapporo Medical Center, Sapporo, Hokkaido, Japan

**Keywords:** Certolizumab pegol, Clinical study, Rheumatoid arthritis, TNFα, TNF inhibitor

## Abstract

*Objectives.* To evaluate the long-term efficacy and safety of certolizumab pegol (CZP) plus methotrexate treatment and to assess the efficacy of two CZP maintenance dosing schedules in Japanese rheumatoid arthritis (RA) patients with an inadequate response to methotrexate.

*Methods.* J-RAPID double-blind patients were entered into an open-label extension (OLE) study. Patients withdrawn due to lack of efficacy at 16 weeks and double-blind completers without a week-24 American College of Rheumatology (ACR) 20 response received CZP 200 mg every other week (Q2W) plus methotrexate. Double-blind completers with week-24 ACR20 responses were randomized to CZP 200 mg Q2W plus methotrexate or CZP 400 mg every 4 weeks plus methotrexate.

*Results*. The ACR20/ACR50/ACR70 response rates of double-blind completers (*n* = 204) were 89.7%/67.2%/36.3% at OLE entry and 95.6%/84.8%/58.3% at 52 weeks, respectively. Other clinical, functional and radiographic outcomes were sustained with long-term CZP plus methotrexate. Long-term treatment with CZP was well-tolerated with no new unexpected adverse events observed. The efficacy and safety of CZP treatment were similar between the two dosing schedules.

*Conclusions*. Continued CZP administration with methotrexate maintained efficacy over 52 weeks and was well-tolerated for Japanese RA patients. No obvious differences in clinical efficacy and safety were observed between the two dosing schedules, giving flexibility in maintenance administration schedules.

## Introduction

TNFα plays a central role in the pathogenesis of rheumatoid arthritis (RA). After the introduction of TNF inhibitors in clinical practice, the management of RA has dramatically changed [1,2]. Early initiation of TNF inhibitors is beneficial not only because they improve the signs and symptoms of RA, but also because they improve physical function and inhibit structural damage, particularly when used in combination with methotrexate (MTX) [3–5]. TNF inhibitors control RA symptoms and suppress functional and structural damages in the long-term, resulting in improved overall outcomes for RA patients [6,7].

In Japan, four TNF inhibitors (infliximab, adalimumab, etanercept and golimumab) have been introduced in clinical practice over the last 10 years [8]. As a relatively new member of the TNFα inhibitor family, certolizumab pegol (CZP) was developed as a novel polyethylene glycolylated (PEG) Fc-free anti-TNFα agent [9,10] and is approved for the treatment of adults suffering from RA not responding to conventional therapy. The efficacy and safety of CZP has been demonstrated in patients with active RA in pivotal international clinical studies [11,12]. In addition, CZP has been shown to improve the signs and symptoms of RA, and decrease disease activity in J-RAPID (concomitant use with MTX) [13] and HIKARI (without MTX) studies performed in Japan [14].

Long-term administration of CZP plus MTX has been previously reported [15], where sustained improvement in RA clinical signs and symptoms including radiographic progression and safety was shown. The aim of the current study was to determine whether the beneficial effects of CZP are sustained during long-term treatment in Japanese RA patients who showed an inadequate response to MTX treatment. To evaluate the long-term efficacy and safety of CZP treatment, we conducted an open-label extension (OLE) study of the J-RAPID study. As a subsidiary objective, we also compared the efficacy of two separate maintenance dosing schedules, CZP 200 mg given every 2 weeks (Q2W) and CZP 400 mg given every 4 weeks (Q4W). We hereby report the 52-week interim results and post-hoc analysis from the ongoing J-RAPID OLE study.

## Materials and methods

### J-RAPID and J-RAPID OLE study design

The J-RAPID OLE study (NCT00851318) is an OLE study of the J-RAPID study (NCT00791999) [13]. In brief, the J-RAPID study [hereinafter referred to as “double-blind (DB) phase”] was a 24-week, Phase II/III, DB study conducted in 66 centers across Japan. Eligible patients were aged from 20 to 74 years and had a diagnosis of RA by the ACR (1987) criteria [16] with at least nine tender and nine swollen joints at screening and baseline. Moreover, the patients must have met at least one of the following criteria at screening: erythrocyte sedimentation rate (ESR) of ≥ 30 mm/hour or C-reactive protein (CRP) of ≥ 1.5 mg/dL. Patients must have received treatment with MTX (with or without folic acid) for ≥ 6 months before study drug administration, with the MTX dose fixed for ≥ 2 months beforehand and within the range of 6–8 mg/week. Patients with extensive comorbidities were excluded from the study. Refer to [13] for detailed inclusion and exclusion criteria of the J-RAPID study. Japanese patients with active RA and an inadequate response to MTX received either CZP or placebo while continuing to receive stable doses of MTX. In the DB phase, the subjects were randomly assigned 1:1:1:1 into four groups: subcutaneous CZP 100, 200 or 400 mg plus MTX, or placebo (saline) plus MTX, every 2 weeks. Patients randomized to CZP plus MTX received induction doses of 200 mg (100 mg group) or 400 mg (200 and 400 mg groups) at weeks 0, 2 and 4. All patients continued to receive MTX at the same dosage taken at DB phase entry (6–8 mg/week). The primary endpoint of this study was an ACR20 response at week 12.

**Figure 1.**
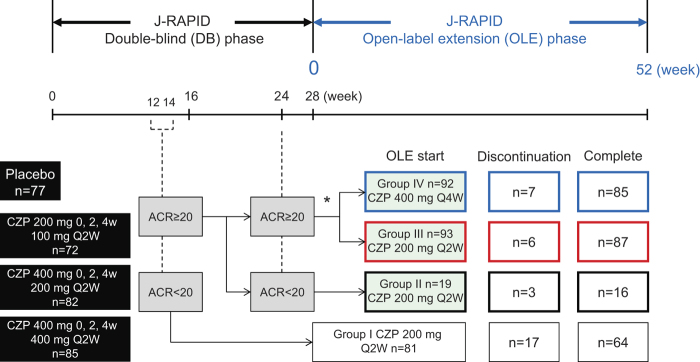
J-RAPID OLE study design. The diagram depicts the breakdown of J-RAPID DB study patients into four groups for the OLE phase of the study. *Regardless of their initial DB phase group assignment, patients who achieved an ACR20 response at weeks 12 or 14 as well as at week 24 were randomized (1:1) to either CZP 200 mg Q2W (Group III, *n* = 93) or CZP 400 mg Q4W (Group IV, *n* = 92).

The J-RAPID OLE study was conducted between April 1, 2009 and August 22, 2011. In the OLE phase, we divided J-RAPID DB phase patients into four groups based on responses to treatment during the DB phase. Patients who did not achieve an ACR20 response at both weeks 12 and 14 were withdrawn from the DB phase at week 16, assigned to Group I (*n* = 81) and treated with CZP 200 mg Q2W plus MTX thereafter. Patients who exhibited an ACR20 response at weeks 12 or 14 but failed to achieve an ACR20 response at week 24 were assigned to Group II (*n* = 19) and also received CZP 200 mg Q2W plus MTX. Patients who achieved an ACR20 response at week 12 or 14 as well as at week 24 were randomized 1:1 to either CZP 200 mg Q2W plus MTX (Group III, *n* = 93) or CZP 400 mg Q4W plus MTX (Group IV, *n* = 92) (Figure 1). Of importance, we established this dosing schedule so that the total dose received by patients in Groups III and IV over a 1-month period was the same.

Week 0 of the OLE phase of Groups II, III and IV (J-RAPID DB phase completers: hereinafter referred to as DB completers) corresponds to week 28 of the DB phase and week 0 of the OLE phase of Group I (early escape) corresponds to week 16 of the DB phase. Patients assigned to the placebo group during the DB phase were also included in this OLE study. Discontinuation of concomitant MTX was not permitted during the OLE phase up to week 52. A change in MTX dosage was permitted after week 24 of the OLE phase, if it was not greater than the original dose (6–8 mg/week).

The outcome of the study was the measurement of continuous efficacy and safety monitoring during long-term treatment with CZP plus MTX. Efficacy outcomes included ACR20 response rates, and changes in Health Assessment Questionnaire Disability Index (HAQ-DI), Disease Activity Score in 28 Joints-Erythrocyte Sedimentation Rate (DAS28-ESR), the Short Form-36 Health Survey (SF-36) and Pain Visual Analog Scale (VAS) from J-RAPID pre-study baseline. In addition, to measure radiographic disease progression, changes in the modified Total Sharp Score (mTSS) from OLE study entry was assessed by linear extrapolation. Comprehensive disease control (CDC) was defined by the simultaneous achievement of the following three criteria: low disease activity (DAS28-ESR ≤ 3.2), functional remission (HAQ-DI ≤ 0.5) and radiographic non-progression (yearly ΔmTSS ≤ 0.5). Similarly, comprehensive disease remission (CDR) was defined by simultaneously achieving the following: clinical remission (DAS28-ESR < 2.6), functional remission (HAQ-DI ≤ 0.5) and radiographic non-progression (yearly ΔmTSS ≤ 0.5). To calculate CDC and CDR for overall DB completers (*n* = 204), yearly ΔmTSS from J-RAPID pre-study baseline (linear extrapolation, with non-responder imputation for patients with no data) was used. Safety outcomes were reported for all patients who received at least one dose of CZP in the OLE study (*n* = 285).

The study was conducted in accordance with the ethical principles of the Declaration of Helsinki and with the Pharmaceutical Affairs Law Standards for the Conduct of Clinical Trials on Drugs (Ministry of Health, Labour and Welfare Ordinance no. 28, 27 March 1997) and related notifications. Institutional review board approval was obtained at all centers and all patients provided written informed consent.

### Post-hoc analyses

Since the OLE study included patients who received placebo during the J-RAPID DB phase, an additional post-hoc analysis of clinical efficacy was performed on patients who received CZP in the DB phase, to observe the effects of continuous CZP treatment during the combined DB and OLE phases of the study. This data set includes patients who were originally assigned to CZP 100, 200 and 400 mg treatment groups in the DB phase and completed the DB phase (CZP-DB completers). We focused on ACR20/ACR50/ACR70 response rates, DAS28-ESR scores, HAQ-DI scores and the disease activity state (high: DAS28-ESR > 5.1, moderate: > 3.2 and ≤ 5.1, low disease activity (LDA): ≤ 3.2 and remission: < 2.6) in this post-hoc analysis.

### Statistical analyses

The efficacy analysis was performed on the full-analysis set (FAS) using the last observation carried forward (LOCF) to impute missing data. We used J-RAPID pre-study parameters as baseline values. Safety analyses were performed on all subjects who received at least one dose of CZP during the OLE study. Because the objective of the study was to evaluate the long-term efficacy and safety of CZP treatment, inferential analyses were not performed.

## Results

### Patient characteristics and disposition of the J-RAPID OLE study

We obtained informed consent from 286 J-RAPID DB phase patients to enter the OLE study. Because one patient withdrew from the OLE study before receiving CZP treatment, a total of 285 patients were included in the efficacy and safety analyses. During the 52-week treatment, an additional 33 patients withdrew from the study resulting in a total of 252 patients (88.4%) completing the 52-week interim period of the OLE study. A few patients (2.5%) withdrew from the study due to an inadequate response (Table 1). All reasons for study withdrawal are listed in Table 1.

**Table 1.** Reasons for discontinuation of therapy.

Subject disposition	Group I CZP 200 mg Q2W	Group II CZP 200 mg Q2W	Group III CZP 200 mg Q2W	Group IV CZP 400 mg Q4W	Total (Groups I + II+ III + IV)
Number of subjects, n (%)*	81 (100.0)	19 (100.0)	93 (100.0)	92 (100.0)	285 (100.0)
Subjects withdrawn before week 52, n (%)*	17 (21.0)	3 (15.8)	6 (6.5)	7 (7.6)	33 (11.6)
Reason for withdrawal					
Subject's request	3 (3.7)	1 (5.3)	1 (1.1)	1 (1.1)	6 (2.1)
Violation of inclusion/exclusion criteria	0 (0.0)	0 (0.0)	0 (0.0)	0 (0.0)	0 (0.0)
Adverse event	7 (8.6)	1 (5.3)	2 (2.2)	5 (5.4)	15 (5.3)
Pregnancy	0 (0.0)	0 (0.0)	1 (1.1)	1 (1.1)	2 (0.7)
Inadequate response	5 (6.2)	1 (5.3)	1 (1.1)	0 (0.0)	7 (2.5)
Compliance with protocol not possible, for reason other than those above	2 (2.5)	0 (0.0)	1 (1.1)	0 (0.0)	3 (1.1)
Investigator's judgment	0 (0.0)	0 (0.0)	0 (0.0)	0 (0.0)	0 (0.0)

*Number of patients (%).

A summary of patient demographics and J-RAPID pre-study baseline characteristics is shown in Table 2. Patients were divided into four groups in the OLE phase based on their response during the DB phase. It was important to distinguish patients with an ACR20 response at week 24 of the DB phase (DB responders: Groups III and IV) and DB non-responders (Groups I and II) to evaluate the sustained efficacy of continued long-term CZP treatment. As shown in Table 2, all of these groups included patients who were on placebo during the DB phase. The fraction of patients that received placebo during the DB phase was 55.6% (45 patients), 31.6% (6 patients), 10.8% (10 patients) and 9.8% (9 patients) in Groups I, II, III and IV, respectively. DB responders were further divided into two groups to evaluate the efficacy of two different dosing regimens. DB responder patients (*n* = 185) were randomized to either CZP 200 mg Q2W (Group III, *n* = 93: 83 CZP patients, 10 placebo patients) or CZP 400 mg Q4W (Group IV, *n* = 92: 83 CZP patients, 9 placebo patients) as shown in Table 2. At OLE study entry (OLE week-0), the mean DAS28-ESR scores of Groups I, II, III and IV were 6.08, 5.12, 3.22 and 3.20, respectively.

**Table 2.** Patient demographics and disease status at J-RAPID pre-study baseline (FAS population).

	Group I	Group II	Group III	Group IV	Total
	CZP 200 mg	CZP 200 mg	CZP 200 mg	CZP 400 mg	(Groups
	Q2W	Q2W	Q2W	Q4W	I + II+ III + IV)
	(*n* = 81)	(*n* = 19)	(*n* = 93)	(*n* = 92)	(*n* = 285)
Prior treatment in the double-blind phase, n (%)*					
Placebo	45 (55.6)	6 (31.6)	10 (10.8)	9 (9.8)	70 (24.6)
CZP 100 mg	14 (17.3)	5 (26.3)	22 (23.7)	24 (26.1)	65 (22.8)
CZP 200 mg	11 (13.6)	5 (26.3)	29 (31.2)	29 (31.5)	74 (26.0)
CZP 400 mg	11 (13.6)	3 (15.8)	32 (34.4)	30 (32.6)	76 (26.7)
Mean age (SD), years	51.8 (10.7)	51.3 (13.7)	52.9 (11.0)	54.1 (11.0)	52.8 (11.1)
Female, n (%)*	67 (82.7)	16 (84.2)	76 (81.7)	77 (83.7)	236 (82.8)
Mean body weight (SD), kg	56.30 (12.00)	54.27 (9.57)	55.81 (11.34)	54.92 (9.47)	55.56 (10.83)
Mean disease duration (SD), years	5.74 (3.94)	5.44 (3.78)	5.94 (4.18)	6.00 (4.16)	5.87 (4.06)
Mean no. of prior DMARDs (SD), excluding MTX	0.8 (0.9)	0.9 (1.0)	0.6 (0.7)	0.5 (0.7)	0.6 (0.8)
Prior TNF inhibitor use, n (%)*	21 (25.9)	4 (21.1)	10 (10.8)	7 (7.6)	42 (14.7)
Mean MTX dose (SD), mg/week	7.5 (0.9)	7.9 (0.5)	7.5 (0.8)	7.5 (0.9)	7.5 (0.8)
RF-positive (≥ 14 IU/mL), n (%)*	73 (90.1)	16 (84.2)	79 (84.9)	83 (90.2)	251 (88.1)
Mean no. (SD) of tender joints (0–68)	21.1 (10.1)	18.4 (11.6)	20.2 (11.7)	18.6 (8.8)	19.8 (10.4)
Mean no. (SD) of swollen joints (0–66)	18.2 (9.2)	17.2 (10.9)	16.3 (8.0)	16.8 (7.9)	17.1 (8.5)
Mean CRP (SD), mg/dL	2.68 (2.40)	2.40 (2.16)	2.03 (1.87)	2.17 (2.13)	2.28 (2.14)
Mean ESR (SD), mm/h	53.6 (24.1)	58.0 (21.3)	49.8 (23.2)	51.5 (21.8)	52.0 (22.9)
DAS28 (ESR)					
Mean (SD)	6.52 (0.77)	6.37 (0.73)	6.22 (0.81)	6.21 (0.83)	
< 3.2, n (%)*	0 (0.0)	0 (0.0)	0 (0.0)	0 (0.0)	0 (0.0)
3.2–5.1, n (%)*	0 (0.0)	1 (5.3)	4 (4.3)	10 (10.9)	15 (5.3)
> 5.1, n (%)*	81 (100.0)	18 (94.7)	89 (95.7)	82 (89.1)	270 (94.7)
HAQ-DI (SD)	1.23 (0.66)	1.03 (0.63)	1.08 (0.63)	1.04 (0.60)	1.11 (0.63)
Mean total mTSS (SD)	46.73 (52.61)	60.41 (46.53)	56.79 (62.23)	53.52 (52.20)	53.10 (55.35)

CRP, C-reactive protein; CZP, certolizumab pegol; DAS28, 28-joint Disease Activity Score; DMARD, disease-modifying antirheumatic drug; ESR, erythrocyte sedimentation rate; FAS, full analysis set; HAQ-DI, Health Assessment Questionnaire – Disability Index; mTSS, modified Total Sharp Score; MTX, methotrexate; RF, rheumatoid factor; SD, standard deviation.

*Number of patients (%).

### Clinical efficacy is sustained by long-term CZP plus MTX treatment

**Figure 2.**
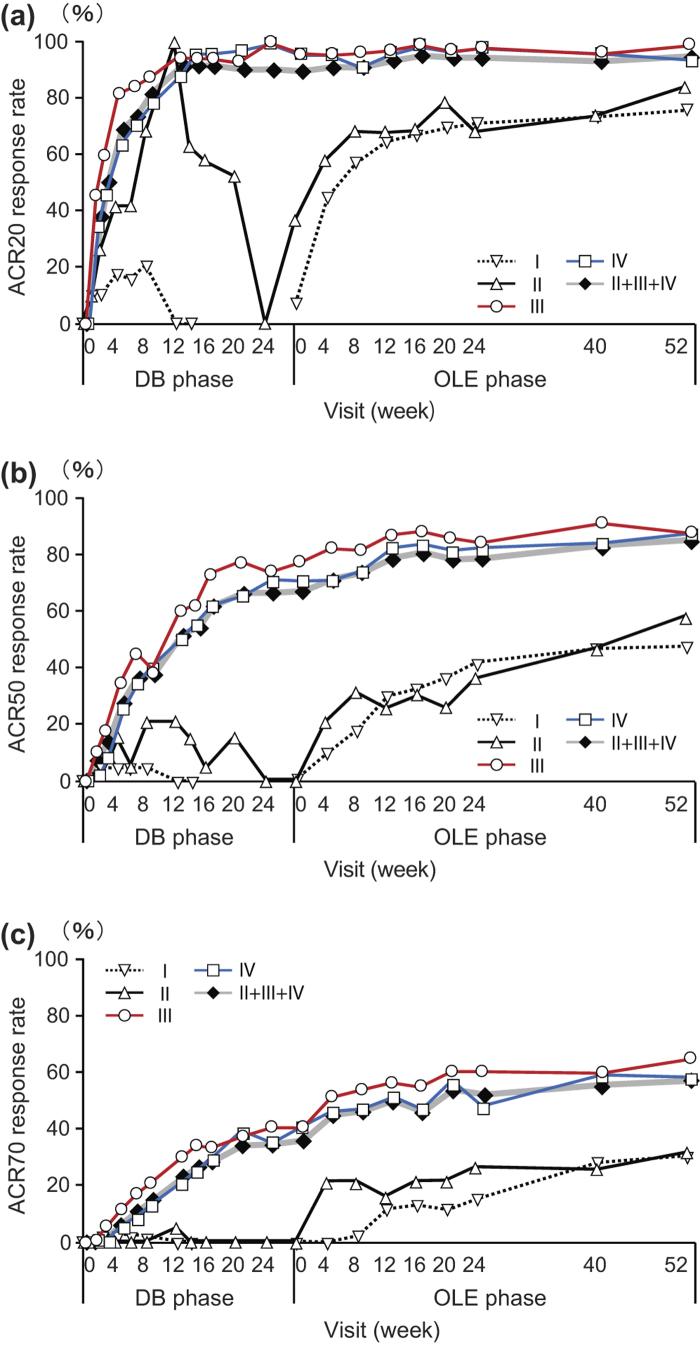
The ACR20/ACR50/ACR70 response rates in patients from each treatment group. The percentages of patients in Groups I (*n* = 81), II (*n* = 19), III (*n* = 93), IV (*n* = 92) and patients in Groups II + III+ IV combined (DB completers, *n* = 204) who achieved an (a) ACR20, (b) ACR50, or (c) ACR70 response were plotted over time for the DB and the OLE phase of the study (FAS population, LOCF imputation). Of note, week 0 of the OLE phase of Group I (early escape) corresponds to week 16 of the DB phase. There are no points in the missing section of the graph for Group I.

**Figure 3.**
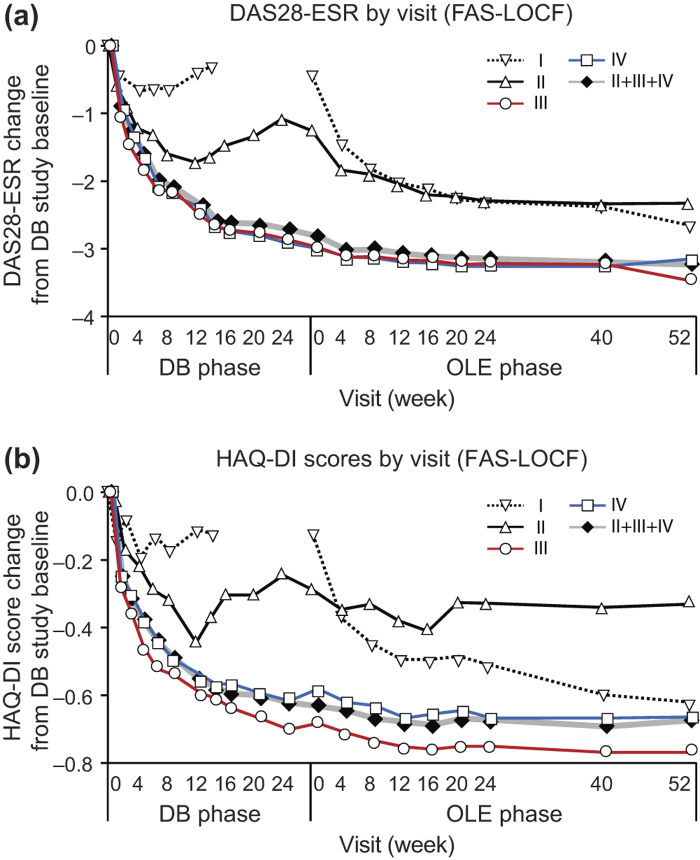
The changes of DAS28-ESR and HAQ-DI over J-RAPID pre-study baseline in patients from each treatment group. Changes in (a) DAS28-ESR and (b) HAQ-DI from J-RAPID pre-study baseline of Groups I (*n* = 81), II (*n* = 19), III (*n* = 93), IV (*n* = 92) and patients in Groups II + III+ IV combined (DB completers, *n* = 204) were plotted against time for the DB and the OLE phase of the study (FAS population, LOCF imputation). Of note, week 0 of the OLE phase of Group I (early escape) corresponds to week 16 of the DB phase. There are no points in the missing section of the graph for Group I.

The J-RAPID OLE study was designed to evaluate whether the benefits obtained after short-term CZP plus MTX treatment could be sustained by prolonged treatment. To this end, we analyzed the outcomes of Groups I, II, III, IV and overall DB completers (Groups II + III+ IV) after up to 52 weeks of long-term CZP treatment in the OLE phase. ACR20/ACR50/ACR70 response rates, calculated from the J-RAPID pre-study baseline, were increased or sustained for up to 52 weeks in the OLE phase. At OLE study entry and at 52 weeks of the OLE phase, the ACR20 response rates were 7.5% and 76.5% for Group I, 36.8% and 84.2% for Group II, 95.7% and 98.9% for Group III, and 94.6% and 94.6% for Group IV, respectively (Figure 2a). The ACR50 response rates were 0% and 48.1% for Group I, 0% and 57.9% for Group II, 77.4% and 87.1% for Group III, and 70.7% and 88% for Group IV, respectively (Figure 2b). The ACR70 response rates were 0% and 30.9% for Group I, 0% and 31.6% for Group II, 39.8% and 64.5% for Group III, and 40.2% and 57.6% for Group IV, respectively (Figure 2c). For overall DB completers (Groups II + III + IV), at OLE study entry and at 52 weeks of the OLE phase, the ACR20 response rates were 89.7% and 95.6%, the ACR50 response rates were 67.2% and 84.8%, and the ACR70 response rates were 36.3% and 58.3%, respectively (Figure 2). A marked improvement in DAS28-ESR was also sustained for up to 52 weeks of the OLE phase for overall DB completers (Figure 3a). DAS28-ESR remission rates (defined as DAS28-ESR < 2.6) for overall DB completers were 28.4% and 42.6% at OLE study entry and 52 weeks of the OLE phase, respectively.

Improvements in quality of life indicators including HAQ-DI (Figure 3b) and SF-36 were maintained by long-term CZP treatment. The HAQ-DI remission rates (defined as HAQ-DI ≤ 0.5) for overall DB completers were 66.7% and 77.5% at OLE entry and at 52 weeks of the OLE phase, respectively, indicating that most patients achieved functional remission. The mean ± SD changes of SF-36 scores from J-RAPID pre-study baseline at OLE study entry and at week 52 were 11.93 ± 10.03 and 13.60 ± 10.54 in physical component summary scores and 5.56 ± 11.07 and 5.21 ± 11.17 in mental component summary scores, respectively. In fact, an individual assessment of all eight domains of the SF-36 revealed sustained improvement in each component of the SF-36 score (data not shown). Moreover, the 100 mm pain VAS improvement was maintained, with a mean ± SD change from J-RAPID pre-study baseline of − 33.8 ± 22.1 at OLE study entry and − 39.2 ± 23.2 at 52 weeks of the OLE phase.

**Figure 4.**
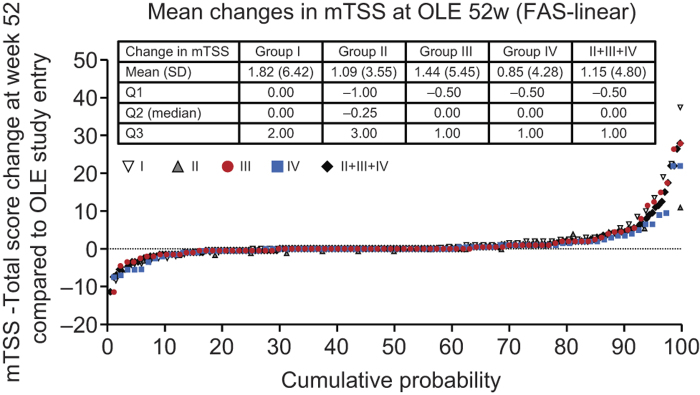
Inhibition of progression of structural damage: cumulative probability plot representing the change from OLE study entry in mTSS at week 52 (FAS population, linear extrapolation). The graph depicts the cumulative probability of patients displaying a particular change in mTSS from OLE study entry in Groups I (*n* = 67), II (*n* = 16), III (*n* = 87), IV (*n* = 83) and patients in Groups II + III+ IV combined (DB completers, *n* = 186).

In addition to signs and symptoms, and patient-reported outcomes, changes from OLE study entry in mTSS (ΔmTSS) were assessed. The mean ± SD and median ΔmTSS at week 52 were 1.15 ± 4.80 and 0.00 in DB completers, respectively (Figure 4). At week 52, 68.3% of DB completers displayed radiographic non-progression, that is a ΔmTSS ≤ 0.5.

The proportion of DB completers achieving comprehensive disease control (CDC: i.e. DAS28-ESR ≤ 3.2, HAQ-DI ≤ 0.5, and ΔmTSS ≤ 0.5) at OLE study entry and at 52 weeks was 25.5% and 35.8%, respectively. The proportion of DB completers achieving comprehensive disease remission (CDR: i.e. DAS28-ESR < 2.6, HAQ-DI ≤ 0.5, and ΔmTSS ≤ 0.5) at OLE study entry and at 52 weeks was 17.2% and 26.0%, respectively. Together, these data suggest that the clinical, functional and radiographic benefits obtained after short-term CZP treatment are sustained by long-term treatment with CZP.

### The two dosing schedules (CZP at 200 mg Q2W vs. 400 mg Q4W) combined with MTX similarly sustain the clinical efficacy of CZP

In both randomized arms (Groups III and IV), the high ACR20/ACR50/ACR70 rates and the high changes from J-RAPID pre-study baseline in DAS28-ESR and HAQ-DI scores were sustained through 52 weeks of the OLE phase (Figures 2 and 3). For example, the ACR20 response rates at OLE study entry and at 52 weeks of the OLE phase were 95.7% and 98.9% for Group III and 94.6% and 94.6% for Group IV, respectively (Figure 2a). In addition to clinical parameters, the ΔmTSS from OLE study entry between the two dosing regimens were similar throughout the OLE study (Figure 4). At week 52, 69.0% and 67.5% of patients had a ΔmTSS ≤ 0.5 in Groups III and IV, respectively, suggesting that both regimens were similarly effective at inhibiting radiographic progression. Thus, both CZP maintenance regimens similarly sustained clinical efficacy of CZP during the OLE study phase.

### Assessment of sustained clinical efficacy of combined long-term CZP plus MTX treatment by a post-hoc analysis through the DB and OLE phase

**Figure 5.**
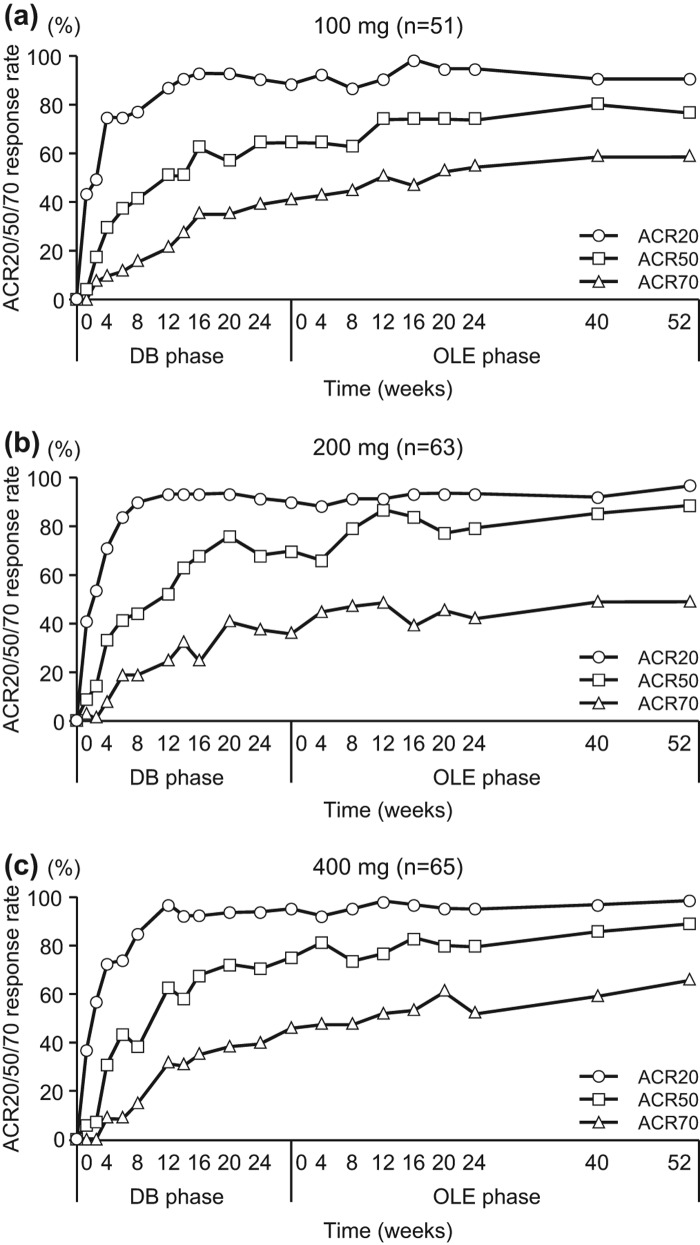
Post-hoc analysis of ACR20/ACR50/ACR70 response rates in patients from Groups II, III and IV excluding those who were in the placebo group during the DB phase (CZP-DB completers). The ACR20, ACR50 and ACR70 response rates of post-hoc analysis patients treated with (a) 100 mg (*n* = 51), (b) 200 mg (*n* = 63) or (c) 400 mg (*n* = 65) of CZP during the DB phase were plotted against time for the DB and the OLE phase of the study (LOCF imputation).

**Figure 6.**
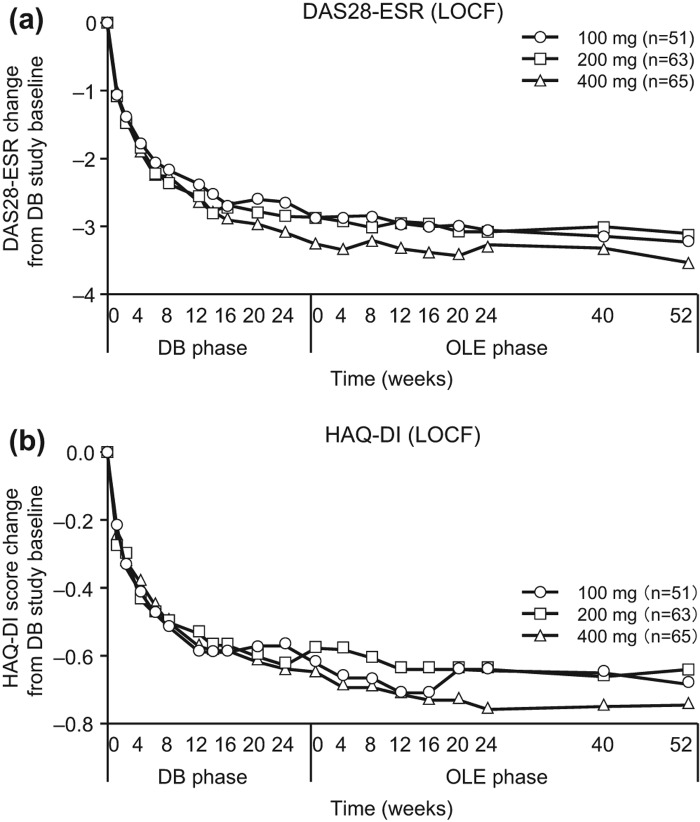
Post-hoc analysis of changes in (a) DAS28-ESR and (b) HAQ-DI scores from J-RAPID pre-study baseline in patients from Groups II, III and IV excluding those who were in the placebo group during the DB phase (CZP-DB completers). The changes of DAS28-ESR and HAQ-DI scores of post-hoc analysis patients treated with 100 mg (*n* = 51), 200 mg (*n* = 63) or 400 mg (*n* = 65) of CZP during the DB phase were plotted against time for the DB and the OLE phase of the study (LOCF imputation).

**Figure 7.**
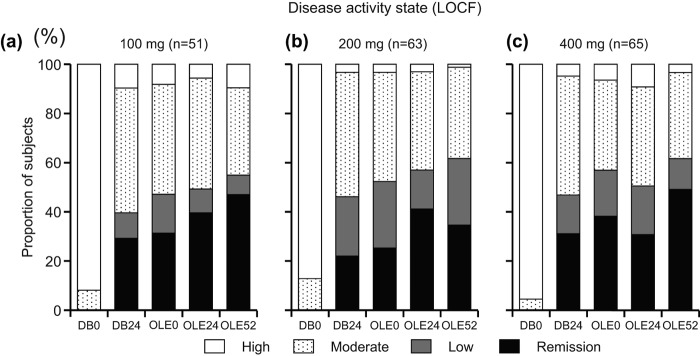
Post-hoc analysis of disease activity states in patients from Groups II, III and IV excluding those who were in the placebo group during the DB phase (CZP-DB completers). The proportions of patients with high (defined as DAS28-ESR > 5.1), moderate (> 3.2 and ≤ 5.1), low (≤ 3.2), or remission (< 2.6) disease activity states at DB week 0 (DB0), DB week 24 (DB24), OLE week 0 (OLE0), OLE week 24 (OLE24) and OLE week 52 (OLE52) among patients treated with (a) 100 mg (*n* = 51), (b) 200 mg (*n* = 63) and (c) 400 mg (*n* = 65) during the DB phase are shown (LOCF imputation).

Since all arms of the OLE protocol (Groups I, II, II and IV) included patients who were originally randomized to the placebo group during the DB phase (Table 2), a post-hoc analysis was performed only on data from patients who were originally assigned to one of the CZP treatment groups in the DB phase and completed the DB phase with an ACR20 response at week 12 or 14 (CZP-DB completers). This was an important analysis to observe the effects of continuous CZP treatment during the DB and OLE phases of the study. In this post-hoc analysis, we focused on ACR20/ACR50/ACR70 response rates, DAS28-ESR scores, HAQ-DI scores and the disease activity state (LDA and remission). The efficacy results are summarized in Figures 5–7 for the patients who were originally randomized to either CZP 100, 200 or 400 mg in the DB phase and received either CZP 200 mg Q2W + MTX (Groups II and III) or CZP 400 mg Q4W + MTX (Group IV) in the OLE phase.

The ACR20/ACR50/ACR70 response rates in CZP-DB completers were sustained with long-term CZP treatment up to week 52 compared with OLE study entry (Figure 5). For example, 87.3% (55/63) of CZP-DB completers receiving 200 mg CZP during the DB phase continued treatment with CZP to 52 weeks of the OLE phase, with the ACR20/ACR50/ACR70 response rates of 96.8%/88.9%/49.2% at week 52, respectively (Figure 5). Moreover, the mean changes in DAS28-ESR scores and HAQ-DI scores, from J-RAPID pre-study baseline, were also sustained up to 52 weeks of the OLE phase (Figure 6a, b). Furthermore, the analysis of disease activity states demonstrated that both LDA and remission rates (defined as DAS28-ESR ≤ 3.2 and < 2.6, respectively) were sustained during the 52-week period of the OLE phase. The proportion of patients who were in LDA and remission (DAS28-ESR ≤ 3.2) was 47.1%, 52.4% and 57.0% at OLE entry, and 54.9%, 61.9% and 61.5% at week 52, in CZP-DB completers receiving 100, 200 and 400 mg CZP during the DB phase, respectively (Figure 7). The remission rates (DAS28-ESR < 2.6) were 31.4%, 25.4% and 38.5% at OLE entry, and 47.1%, 34.9% and 49.2% at week 52, in CZP-DB completers receiving 100, 200 and 400 mg CZP during the DB phase, respectively (Figure 7). Therefore, this post-hoc analysis demonstrates that the efficacy of CZP can be sustained in long-term CZP treatment, even when the analysis set is restricted to patients who have achieved an ACR20 clinical response after 12–14 weeks of CZP treatment.

### Adverse events (AE)s reported during long-term CZP plus MTX treatment

During the 52 weeks of the OLE phase, 253 patients (88.8%) experienced AEs and 31 patients (10.9%) experienced serious AEs (Table 3). Among SAEs, two patients (0.7%) exhibited abnormal hepatic function, two patients (0.7%) developed bronchitis, three patients (1.1%) displayed RA exacerbations and two patients (0.7%) developed subarachnoid hemorrhage. Two patients (0.7%) developed a malignancy (breast cancer, colon cancer). The most common AEs were nasopharyngitis, pharyngitis and upper respiratory tract infections. Most AEs were mild to moderate (84.6%). The rate, severity and distribution of AEs were similar among all groups (Groups I–IV), suggesting that no obvious differences in AEs are observed based on the CZP treatment schedule. No tuberculosis infections or deaths were reported. No unanticipated AEs occurred in any of the groups. Thus, over 52 weeks of the OLE phase, CZP coadministered with MTX was well tolerated, with no new safety precautions when compared with the DB phase (Table 3).

**Table 3.** Treatment-emergent adverse events.

	Group I	Group II	Group III	Group IV	Total
	CZP 200 mg	CZP 200 mg	CZP 200 mg	CZP 400 mg	(Groups
	Q2W	Q2W	Q2W	Q4W	I + II+ III + IV)
	(*n* = 81)	(*n* = 19)	(*n* = 93)	(*n* = 92)	(*n* = 285)
Any adverse event, n (%)*	72 (88.9)	16 (84.2)	83 (89.2)	82 (89.1)	253 (88.8)
Intensity^#^, n (%)*					
Mild	36 (44.4)	6 (31.6)	39 (41.9)	41 (44.6)	122 (42.8)
Moderate	34 (42.0)	8 (42.1)	43 (46.2)	34 (37.0)	119 (41.8)
Severe	2 (2.5)	2 (10.5)	1 (1.1)	7 (7.6)	12 (4.2)
Treatment-related^a^	36 (44.4)	7 (36.8)	43 (46.2)	44 (47.8)	130 (45.6)
Death, n (%)*	0 (0.0)	0 (0.0)	0 (0.0)	0 (0.0)	0 (0.0)
Most common adverse events (≥ 5% in any group), n (%)*					
Nasopharyngitis	20 (24.7)	7 (36.8)	28 (30.1)	28 (30.4)	83 (29.1)
Pharyngitis	8 (9.9)	0 (0.0)	8 (8.6)	7 (7.6)	23 (8.1)
Upper respiratory tract infection	12 (14.8)	2 (10.5)	10 (10.8)	10 (10.9)	34 (11.9)
Contusion	7 (8.6)	0 (0.0)	7 (7.5)	2 (2.2)	16 (5.6)
RA	3 (3.7)	3 (15.8)	4 (4.3)	7 (7.6)	17 (6.0)
Eczema	5 (6.2)	2 (10.5)	7 (7.5)	5 (5.4)	19 (6.7)
Hypertension	4 (4.9)	1 (5.3)	6 (6.5)	6 (6.5)	17 (6.0)
Serious adverse events, n (%)*	9 (11.1)	3 (15.8)	7 (7.5)	12 (13.0)	31 (10.9)
Serious adverse events (≥ 0.5% in any group), n(%)					
Hepatic function abnormal	1 (1.2)	0 (0.0)	0 (0.0)	1 (1.1)	2 (0.7)
Bronchitis	1 (1.2)	1 (5.3)	0 (0.0)	0 (0.0)	2 (0.7)
RA	0 (0.0)	1 (5.3)	0 (0.0)	2 (2.2)	3 (1.1)
Subarachnoid hemorrhage	0 (0.0)	0 (0.0)	0 (0.0)	2 (2.2)	2 (0.7)

*Number of patients (%).

^a^Treatment-emergent adverse events for which the relationship to the study drug cannot be ruled out.

^#^The severity of an adverse event was assessed according to the following three categories.

1) Mild: An event that caused discomfort, but did not interfere with daily activities.

2) Moderate: An event that was sufficiently discomforting to restrict or interfere with daily activities.

3) Severe: An event that prevented work or daily activities.

## Discussion

The 24-week treatment of CZP has been shown to be efficacious in improving RA disease activity [11–14]. This was also true in patients who showed an inadequate response to MTX. In the DB placebo-controlled J-RAPID study, the combination of CZP plus MTX for 24 weeks improved disease outcome in RA patients who showed an inadequate response to MTX [13]. Although several data on short-term CZP plus MTX treatment have been available [17–19], the clinical efficacy and safety of long-term CZP plus MTX treatment is unknown in Japanese RA patients. Thus, we conducted an OLE study of the J-RAPID study to evaluate the safety of combined long-term CZP plus MTX treatment and to investigate whether the clinical benefit obtained from the 24-week DB phase of the J-RAPID study could be sustained by extending the treatment for another 52 weeks. In addition, we used the OLE herein to assess the efficacy of two different maintenance dosing schedules, the standard dosing (CZP 200 mg Q2W) and an alternative regimen (CZP 400 mg Q4W), both with concomitant MTX.

Our data demonstrate that long-term CZP treatment sustains the clinical efficacy obtained in overall DB completers. This was true for ACR response rates, DAS28-ESR, SF-36 scores and the pain VAS. In addition, clinical remission was observed in 42.6% of patients with long-term treatment at 52 weeks of the OLE study. Functional remission was also observed in 77.5% at 52 weeks of the OLE. Moreover, an analysis of mTSS scores showed that radiographic non-progression (ΔmTSS ≤ 0.5) was achieved in 68.3% of the patients. In terms of the radiographic scores, as described in the Materials and Methods, all patients received CZP in this OLE, and the changes in mTSS scores were within the 52 weeks of the OLE. Therefore, there are no significant differences seen in radiographic progression between groups. On a separate note, the disconnect between the improvements in signs and symptoms and changes in radiographic manifestations of disease has been previously reported in patients treated with TNF antagonists [20].

Together, these results suggest that long-term CZP treatment is effective at controlling RA disease progression, even with the relatively low dose of concomitant MTX (6–8 mg/week). The low withdrawal rate (2.5%) of patients from the study due to lack of efficacy further supports this notion. This was true even for patients that were initially treated with the lower dose of CZP (100 mg) during the DB phase. Although the rate of LDA was approximately 7% lower in patients treated with 100 mg CZP during the DB phase compared with patients treated with 200 mg CZP at 52 weeks of the OLE phase, there were no overall significant differences in disease activity state regardless of the dose received during the DB phase (Figure 7).

This is the first study that demonstrates the benefits of continued long-term treatment of CZP administration in Japanese RA patients. Similar studies have been conducted internationally. In the RAPID1 trial, sustained benefit in clinical signs and symptoms and radiographic progression was observed after 2 years of continuous CZP treatment [15]. More recently, the 5-year OLE study from the RAPID1 trial showed continued efficacy up to 256 weeks with no new safety signals identified [21]. Our data presented here suggest that Japanese patients continue to receive relief from RA symptoms after long-term TNFα inhibition by CZP. Importantly, long-term CZP plus MTX treatment was well-tolerated as no unexpected new AEs were detected in patients compared with those observed in previous clinical studies involving short-term CZP plus MTX treatment.

CZP is a novel anti-TNFα monoclonal antibody consisting of a humanized Fab’ fragment fused to a 40-kD PEG moiety [22,23]. One drawback of Fab’ fragments is that the clearance of Fab’ fragments is accelerated in the absence of the Fc region, leading to shorter *in vivo* half-lives compared with full antibodies. However, by attachment of the PEG moiety to the Fab’ fragment, the plasma half-life of CZP was extended to about 2 weeks. Because of the extended half-life, a more spaced out CZP maintenance dosing schedule is possible. Our current study provides evidence that extending the interval to Q4W for CZP maintenance therapy is as effective as the Q2W regimen. No obvious differences in clinical efficacy and safety were observed between patients treated with CZP 200 mg Q2W and CZP 400 mg Q4W (Group III vs. Group IV). Thus, patients and physicians have the flexibility of choosing either of two maintenance dosing schedules based on their needs. For example, a Q4W dosing schedule might decrease the number of doctor visits and thus, might be an attractive option for some patients.

The design of the J-RAPID OLE study included patients that were previously on placebo during the DB phase of the J-RAPID study. To observe the effects of continuous CZP treatment through the DB and OLE phases of the study (80 weeks), an additional post-hoc analysis was performed on CZP-DB completers (Groups II–IV) who received CZP during the DB phase. Restricting our analysis to these patients clearly showed that long-term CZP plus MTX treatment sustained the clinical, radiographic and functional efficacy against disease. Thus, we conclude that long-term CZP plus MTX treatment for up to 80 weeks results in a sustained positive response.

Administration of MTX remains the cornerstone for treatment of RA [24,25]. However, some patients do not achieve the desired response when MTX is used as monotherapy [25]. Thus, it is important to identify drugs that can be used in conjunction with MTX to more effectively treat the symptoms of RA. The J-RAPID study demonstrated that CZP is clinically effective in combination with MTX for treatment of patients who failed to achieve a satisfactory response with MTX alone. Our current study is the first to investigate the clinical efficacy of CZP with MTX treatment over an ˜80-week period (28 weeks during the DB phase + 52 weeks during the OLE phase) in Japanese RA patients. Our data demonstrate that long-term CZP plus MTX treatment sustains the beneficial effect of CZP plus MTX afforded after 24 weeks of therapy. Moreover, no new unexpected AEs were discovered during the OLE phase, suggesting that additional risks are not incurred by long-term treatment with CZP plus MTX. Based on results from clinical trials and from post market surveys, the AEs observed with long-term CZP treatment are comparable to those seen with other TNF inhibitors such as infliximab, etanercept, adalimumab and golimumab. As an added benefit, the local skin reaction to subcutaneous injection of CZP tends to be lower than other subcutaneously administered TNF inhibitors. Together, these data suggest that if the patient obtains a positive response to CZP treatment after 12–14 weeks, clinicians can expect sustained efficacy without additional risks by continuing onto long-term use of CZP plus MTX.

In summary, our data suggest that continuous long-term CZP treatment is a beneficial option in patients with active RA and an inadequate response to MTX, by providing long-term clinical, functional and radiographic disease control. This was true for both the Q2W and Q4W maintenance dosing schedules of CZP. Moreover, long-term treatment was well-tolerated with no new unexpected adverse events observed. One limitation of our study was that this was an OLE study and therefore not blinded. However, we believe that our data still suggest that long-term CZP treatment is beneficial for continued suppression of RA. Thus, we propose that patients with active RA and an inadequate response to MTX should undergo continuous combined long-term treatment with either a CZP 200 mg Q2W or CZP 400 mg Q4W schedule with MTX to achieve long-lasting suppression of RA symptoms.
